# High body fat percentage and low consumption of dairy products were associated with vitamin D inadequacy among older women in Malaysia

**DOI:** 10.1371/journal.pone.0228803

**Published:** 2020-02-13

**Authors:** Kok Hong Leiu, Yit Siew Chin, Zalilah Mohd Shariff, Manohar Arumugam, Yoke Mun Chan

**Affiliations:** 1 Department of Nutrition and Dietetics, Faculty of Medicine and Health Sciences, Universiti Putra Malaysia, Serdang, Selangor, Malaysia; 2 Research Centre of Excellence, Nutrition and Non-Communicable Diseases, Faculty of Medicine and Health Sciences, Universiti Putra Malaysia, Serdang, Selangor, Malaysia; 3 Department of Orthopedics, Faculty of Medicine and Health Sciences, Universiti Putra Malaysia, Serdang, Selangor, Malaysia; Addis Ababa University School of Public Health, ETHIOPIA

## Abstract

**Background:**

Serum vitamin D insufficiency is a public health issue, especially among older women. Sun exposure is fundamental in the production of vitamin D, but older women have less optimal sun exposure. Therefore, factors such as body composition and diet become more essential in sustaining sufficient serum levels of vitamin D. The objective of the current study is to determine factors contributing towards serum vitamin D insufficiency among 214 older women.

**Methods:**

The respondents had their body weight, height, waist circumference and body fat percentage measured, as well as interviewed for their socio-demographic characteristics, sun exposure and dietary intake. Fasting blood samples were obtained from the respondents to measure their serum 25-hydroxyvitamin D [25(OH)D] concentration.

**Results:**

There were 82.7% (95% CI: 77.6%, 87.8%) of the respondents that had serum vitamin D insufficiency (< 50 nmol/L) with an average of 37.4 ± 14.3nmol/L. In stepwise multiple linear regression, high percentage of body fat (ß = -0.211, p <0.01) and low consumption of milk and dairy products (ß = 0.135, p <0.05) were the main contributors towards insufficient serum vitamin D levels, but not socio-demographic characteristics, other anthropometric indices, sun exposure and diet quality.

**Conclusion:**

Older women with high body fat percentage and low dairy product consumption were more likely to have serum vitamin D insufficiency. Older women should ensure their body fat percentage is within a healthy range and consume more milk and dairy products in preventing serum vitamin D insufficiency.

## Introduction

Vitamin D is an important vitamin needed to regulate the homeostasis of calcium and phosphorus in the body [1]. Vitamin D also involved in cell differentiation and anti-proliferation in many cell types, such as muscle, skin, bone marrow and intestine [2]. However, people around the world, especially among older adults in Malaysia are experiencing insufficient vitamin D [3], which is commonly defined as having serum 25(OH)D concentration below 50 nmol/L (20 ng/mL) [4]. As a result, adults with insufficient vitamin D are at a higher risk of developing osteoporosis, muscle weakness, falls and fractures at the hip and vertebral [5,6]. Also, a previous study reported that older women with insufficient vitamin D had a significant reduction in physical and cognitive function [7].

Sun exposure is linked with serum 25(OH)D, especially among older adults aged 60 years and above [8,9]. Sun exposure for 5 to 15 minutes twice or thrice a week [10] allows the skin to generate about 80% of the vitamin D required by the human body [11]. Older adults rarely expose themselves to the sun nowadays, often using sunscreen when outdoors or they stay indoors due to the rising temperature of the earth [12]. Consequently, avoiding being exposed to the sun reduces the possibility for the skin to generate sufficient vitamin D that the body requires [8], especially older adults with thinner skin that has less vitamin D precursors under the skin and reduces the efficiency in vitamin D synthesis [5,13]. Therefore, it is essential to determine the level of average sun exposure received and the impact on serum 25(OH)D among older adults.

Diet is another factor that is closely related to serum 25(OH)D of older adults. In the past, there have been limited choices of foods high in vitamin D, for example, fatty fish, shitake mushrooms and egg yolk for consumption [14]. With the advancement of food technology, there are now foods that are fortified with vitamin D, such as milk and dairy products and breakfast cereals to provide alternatives for people to choose and improve their vitamin D status [15]. Although there are more foods available which are high in vitamin D, the worldwide prevalence of vitamin D inadequacy is still high [3]. This situation might have resulted from a low intake of vitamin D rich food, such as milk and dairy products as reported in the previous Malaysian study [16]. Besides, older adults are encouraged to consume vitamin D rich foods from different sources [17]. Diet quality indices can be utilized to determine whether older adults are consuming foods from different sources based on the recommendations stated in the dietary guidelines [18], and the contribution of diet quality towards vitamin D status for optimal biological functioning within the body.

A prior review reported that obesity and vitamin D inadequacy were closely related to each other [19]. Older adults undergo changes in the body composition around the age of 60 years as muscle is lost, often more fat mass is gained resulting in a lower metabolism rate [20,21]. As a result, any extra fat mass gained can become a storage site for the fat-soluble vitamin D in the body [22], which leads to less vitamin D being released into the blood circulation [19]. There are possibilities that the ongoing changes in body composition among older adults and different obesity indicators make the relationship between different anthropometric indices, such as body mass index (BMI), waist circumference and body fat percentage with vitamin D status to be inconsistent [23–26]. Therefore, it is essential to consider various anthropometric indices that can be utilized to estimate vitamin D status among older adults.

Sun exposure, diet and anthropometric indices, such as body fat percentage, BMI and waist circumference may have an association with vitamin D status among older adults. Past Malaysian studies conducted were focused on the relationship of a single factor, such as adiposity [25], sun exposure [27] and calcium intake [28] with vitamin D status. However, limited Malaysian studies that determine which factor contributes the most to insufficient serum 25(OH)D among older adults, especially women who are prone to bone-related fractures in Malaysia [29]. Hence, the current study determines the factors that contribute to vitamin D inadequacy among older women in Malaysia.

## Methods

### Study design and participants

This cross-sectional study involved older women aged 50 years and above who were menopausal for at least five years or more. The current study was approved by the Ethics Committee for Research involving Human Subjects (JKEUPM), Universiti Putra Malaysia [FPSK(FR16)P017]. The sample size was calculated using the formula for correlation [30] and G*Power software [31] under the statistical test of linear multiple regression: fixed model, R^2^ increase. The highest sample size calculated was 193, while an adjustment of 10% was made to account for the respondents who are deceased or moved out from the residential area when the name list was being compiled [32]. As a result, a minimum sample size of 214 postmenopausal women was required in the present study.

In terms of sampling, there are a total of 15 affiliates under the National Council of Senior Citizens Organisations Malaysia (NACSCOM) in Kuala Lumpur and Selangor. All 15 affiliates were approached, with only seven of the affiliates providing consent to the researchers to conduct the study. The remaining eight affiliates were unable to do so due to several reasons, such as a packed program schedule, no interest in the study, a small number of members, and cannot reach the person-in-charge.

Next, the study information was given to the president of the consented affiliates to be shared with their members. There were 283 women consented for screening. During the screening, respondents were excluded if they had hormone replacement therapy, or any evidence of diagnosed medical conditions, such as cardiovascular diseases, damaged liver or renal disease. After screening, 214 older Chinese women met the inclusion criteria and provided their written informed consent to participate in the study.

### Socio-demographic characteristics

The data collectors interviewed the respondents face-to-face using the questionnaire to obtain demographic information, such as age, years of menopause, marital status, educational level, working status and monthly household income.

### Anthropometric measurement

All the measurements were taken according to the standard of the International Society for the Advancement of Kinanthropometry (ISAK) [33]. Body weight and height of the respondents were measured using TANITA digital weighing scale HD306 to the nearest 0.1 kg and portable SECA stadiometer to the nearest 0.1 cm, respectively. The BMI of the respondents were determined using the body weight with height in meter squared, then categorized as body weight status based on the WHO criteria [34].

The waist circumference of the respondents was measured using a Lufkin measuring tape W606PM to the nearest 0.1 cm, while their body fat percentage was measured through bio-electrical impedance using handheld OMRON portable body fat analyser. The respondents need to hold the body fat analyser with both hands and arms straightened forward. The percentage of body fat is calculated based on the input of individual data, such as age, height and weight [35]. Respondents who had a waist circumference of 80cm and above were categorized with abdominal obesity [36], whereas respondents who had body fat percentage more than 31% were considered as having high body fat [37].

### Sun exposure

Sun exposure of the respondents was determined using the Sun Exposure Index (SEI) [38]. The researchers interviewed the respondents using a daily log developed by Hall and colleagues [39] to recall the duration and location of outdoor activities, usage of sunscreen as well as clothing worn from 7.00 am to 7.00 pm in a typical week as in tropical climate seasons. Body surface area (BSA) of the respondents with sunlight exposure was assessed using an adapted “rule of nines” and clothing worn throughout the day [40]. The SEI of the respondents were then calculated by multiplying the duration of sun exposure every week in minutes with the BSA exposed to sunlight. The higher the SEI score, the higher the amount of sun exposure. However, the sun exposure measured in the current study does not include the assessment of UV index. Based on the report from WHO [41], the maximum UV index reported in tropical countries, such as Singapore and Malaysia ranged from 10 to 13 throughout the year.

### Diet quality

The researcher interviewed the respondents to report the frequency and serving size of 165 food items consumed last month using a semi-quantitative FFQ, adapted from the National Health Morbidity Survey (NHMS) 2014: Malaysian Adult Nutrition Survey (MANS) [42]. The serving size of each food item was determined using the Malaysian Food Album [43]. The amount of food items consumed were converted into weight in grams and entered into the NutritionistPro™ software (First DataBank Corporation, Axxya Systems, Stafford, TX, USA) for analysis.

Next, the diet quality of the respondents was determined by using the Healthy Eating Index (HEI) for Malaysians [44], which covers nine components. The first seven components evaluate the compliance to the recommended intakes as outlined in the Malaysian Dietary Guidelines (MDG) [18] for the following food groups: cereals and grains, vegetables, fruits, poultry, meat and eggs, fish and seafood, legumes, as well as milk and dairy products. The last two components evaluate the compliance to the recommended intake of sodium and percentage of fat from the total energy intake as stated in the Recommended Nutrient Intakes (RNI) for Malaysia [45].

The response for each component was given zero points for no compliance and ten points for total compliance, whereas the points were distributed proportionately for those in-between responses. A composite point was calculated using the formula: total score of nine components / 9 × 10) × 100%. The overall diet quality among the respondents was a calculated composite point score. The higher point score indicates better diet quality and higher compliance with the recommended intake recommended in the MDG and RNI.

### Serum 25(OH)D concentration

All 214 respondents fasted overnight for a minimum of eight hours before the morning blood collection. A certified phlebotomist collected 5ml of venous blood samples from each respondent according to the WHO guidelines on blood drawing [46] and transferred into test tubes to be sent to a commercial laboratory for analysis. The serum 25(OH)D concentrations were determined using the Siemens Advia Centaur XP immunoassay system (Siemens, Munich, Germany). The serum 25(OH)D concentration of each respondent was classified into three different categories: deficiency (< 30 nmol/L), insufficient (30 to < 50 nmol/L) and sufficient (≥ 50 nmol/L) according to the cut-off recommended by the Institute of Medicine [4].

### Data collection and quality management

The data collectors recruited in the current study were postgraduate students with nutrition and health sciences background. The researchers of the current study conducted a briefing and training on the administration of the questionnaire and anthropometric measurements. The researchers also conducted a pre-test in the Institute of Gerontology, Universiti Putra Malaysia to test the understanding and ability of the postmenopausal women to provide appropriate responses based on the questionnaire.

### Statistical analysis

Data were analysed using IBM SPSS Statistics version 22.0 (IBM Corp., Armonk, NY, USA). Descriptive statistics were used to present socio-demographic characteristics, anthropometric measurements, sun exposure, diet quality and serum 25(OH)D concentrations. Bivariate analysis, such as Pearson product-moment correlation, independent-samples t-test, one-way ANOVA and simple linear regression were utilised. Variables with *p* < 0.25 in the simple linear regression [47], such as BMI, waist circumference, body fat percentage, fruit intake, dairy intake and HEI score were entered in the stepwise multivariate model to determine the contribution of socio-demographic characteristics, anthropometric measurements, sun exposure and diet quality to serum 25(OH)D concentrations.

A valid linear regression analysis should fulfil statistical assumptions, including no perfect multicollinearity, regression model fit and normally distributed data [48]. Multicollinearity was determined based on tolerance and variance inflation factor (VIF). When tolerance value was less than 0.1 or VIF value more than 10, multicollinearity is present [49]. The fitness of the regression model is determined based on the significant F value in the ANOVA table, which should be less than 0.05. Normality of all continuous data was determined based on the skewness test [50]. Non-normally distributed data, such as consumption of milk and dairy products were transformed using logarithm data transformation. The statistical significance level was set at *p* < 0.05.

## Results

In the present study, the average age of the respondents was 67.2 ± 6.6 years old, with a mean of 16.1 ± 7.8 years of menopause. The majority of the respondents were married (77.6%), had their education up to secondary level (40.7%), retired (49.5%) and originated from a low monthly household income group (44.4%) (Table 1). The respondents had a mean BMI of 24.4 ± 4.0 kg/m^2^, whereby approximately two out of five respondents (38.8%) were in the overweight and obese categories. The respondents had a mean waist circumference of 80.2 ± 8.9 cm, and almost half of them (48.6%) had abdominal obesity. The respondents had a mean of 35.1 ± 5.2% body fat percentage, whereby 82.2% of them had an unhealthy body fat percentage (Table 1). The respondents had a median of 180.0 minutes of sun exposure per week, with a median SEI of 36.3. The respondents had a mean serum 25(OH)D concentration of 37.4 ± 14.3 nmol/L, whereby more than three-quarters of them (82.7%) had insufficient serum 25(OH)D level (Table 1).

**Table 1:** Characteristics of the respondents (n = 214).

Variable	n (%)	Mean ± SD / Median (IQR)
Age		67.2 ± 6.6
Years of menopause		16.1 ± 7.8
Marital status		
Single	20 (9.3)	
Married	166 (77.6)	
Divorced	6 (2.8)	
Widowed	22 (10.3)	
Educational level		
No formal education	26 (12.1)	
Primary education	71 (33.2)	
Secondary education	87 (40.7)	
Tertiary education	30 (14.0)	
Working status		
Working	28 (13.1)	
Housewife	80 (37.4)	
Retiree	106 (49.5)	
Monthly household income #		
Low: ≤ RM 2300	95 (44.4)	
Middle: RM 2300—RM 5599	79 (36.9)	
High: ≥ RM5600	40 (18.7)	
BMI (kg/m^2^)		24.4 ± 4.0
BMI category		
Underweight	10 (4.7)	
Normal	121 (56.5)	
Overweight	68 (31.8)	
Obesity	15 (7.0)	
Waist circumference (cm)		80.2 ± 8.9
Waist circumference category		
Acceptable: < 80 cm	110 (51.4)	
Unhealthy: ≥ 80 cm	104 (48.6)	
Body fat percentage (%)		35.1 ± 5.2
Body fat percentage category		
Acceptable: 9–31%	38 (17.8)	
Unhealthy: ≥ 32%	176 (82.2)	
Duration of sun exposure per week (min)		180.0 (60.0, 300.0) *
SEI		36.3 (13.8, 60.0) *
Serum 25(OH)D (nmol/L)		37.4 ± 14.3
Serum 25(OH)D Level		
Deficiency: < 30 nmol/L	71 (33.2)	
Insufficient: 30 - < 50 nmol/L	106 (49.5)	
Sufficient: ≥ 50 nmol/L	37 (17.3)	
HEI for Malaysians composite score		66.9 ± 9.9

*Median value (IQR) was reported as data were not normally distributed.

^#^Based on the classification by Economy Planning Unit, Prime Minister’s Department of Malaysia.

There were 36.0% and 31.3% of the respondents with insufficient serum 25(OH)D level had low and medium monthly household income, respectively. Despite having a high household income, there was still 15.4% of the respondents with insufficient serum 25(OH)D level. On the other hand, there were less than one-tenth of the respondents with sufficient serum 25(OH)D level across different monthly household income group, from low (8.4%), medium (5.6%) and high (3.3%).

The respondents had an average energy intake of 1360 ± 468 kcal. The respondents had a sufficient percentage of energy intake from carbohydrates (61.5 ± 7.7%) and protein (20.1 ± 4.7%), while they had a lower percentage of energy intake from fat (18.5 ± 5.0%) compared to the recommended range stated in the RNI for Malaysia. Regarding food group and nutrients intake, the respondents consumed sufficient servings of cereal and grains (4.0 ± 1.7 servings), but they did not consume sufficient servings of other food groups, such as vegetables (2.5 ± 1.6 servings), fruits (1.9 ± 1.7 servings), fish and seafood (0.9 ± 0.9 serving), legumes (0.4 ± 0.4 serving) as well as milk and dairy products [0.1 (0.0, 0.5) serving]. The sodium intake [11.57.4 (838.5, 1689.3) mg] among the respondents was within the recommended range stated in the MDG. Meanwhile, the respondents over-consumed poultry, meat and eggs (1.6 ± 1.0 servings) as compared to the recommended range of ½ to 1 serving stated in the MDG. Therefore, the mean HEI for Malaysian composite score was 66.9 ± 9.9 (Table 1).

Through simple linear regression, two variables were significantly associated with serum 25(OH)D concentrations, which were the percentage of body fat (*β* = - 0.218, *p* < 0.01) and the consumption of milk and dairy products (*β* = 0.146, *p* < 0.05). The socio-demographic characteristics, BMI, waist circumference, sun exposure, intake of other food groups and diet quality were not significantly associated with serum 25(OH)D concentrations (Table 2). Meanwhile, BMI was significantly correlated with waist circumference (*r* = 0.854, *p* < 0.001) and percentage of body fat (*r* = 0.760, *p* < 0.001). Waist circumference was also significantly correlated with the percentage of body fat (*r* = 0.695, *p* < 0.001). Despite having a high correlation between the anthropometric indices, the result did not violate the assumption of high collinearity in the stepwise multiple linear regression.

**Table 2:** Simple linear regression.

Variable	Range	R^2^	Unstandardised coefficients	*p-*value
			B	
BMI (kg/m^2^)	16.2–44.1	0.017	− 0.459	0.059
Waist circumference (cm)	58.4–108.9	0.010	− 0.164	0.136
Body fat percentage (%)	11.3–48.3	0.048	− 0.603	0.001
Fruits (serving)	0.0–8.6	0.010	− 0.829	0.149
Log dairy (serving)	0.0–0.6	0.021	16.163	0.032
HEI for Malaysians composite score	39.3–95.7	0.010	0.144	0.145

Only variables with a p-value less than 0.25 were included.

For the stepwise multiple linear regression analysis (Table 3), the same two variables, body fat percentage and intake of milk and dairy products significantly explained about 6.6% of the variances in the concentrations of serum 25(OH)D of the respondents. The high body fat percentage and low intake of milk and dairy products contributed to low serum 25(OH)D concentrations among the respondents.

**Table 3:** Determinants of vitamin D status from a stepwise multiple linear regression.

Variable	Unstandardised coefficients	Standardised coefficients	ΔR^2^	t	*p*-value
	**B**	***ß***			
Constant	56.372			6.618	0.0001
Body fat percentage	− 0.584	− 0.211	0.048	− 0.211	0.002
Log milk and dairy products (serving)	14.922	0.135	0.018	0.135	0.044

R^2^ = 0.066, Adjusted R^2^ = 0.057, *F* (2, 211) = 7.452, *p* < 0.01.

## Discussion

Approximately four out of five respondents (82.7%) in the present study had insufficient vitamin D. The prevalence was similar to a past local study involving women aged 45 years and above in Kuala Lumpur [27]. Conversely, there are earlier studies with a lower prevalence in vitamin D insufficiency among older women in Malaysia [28,51–53] and women aged 25 to 60 years in Singapore [54]. The differences in the prevalence of insufficient vitamin D might be attributed to the demographic factor. A past local study reported that women living in the rural area had more sun exposure as well as higher vitamin D status compared to those living in an urban area [27].

The main association to the vitamin D level among the respondents was body fat percentage instead of BMI and waist circumference. Aging promotes fat deposition [21] and fat redistribution within the body at various locations, such as abdomen, waist and hip [55]. Also, adipose tissue within the body is the primary storage site of fat-soluble vitamin D [56] and expresses vitamin D receptors (VDR) together with enzymes involved in vitamin D metabolism [57]. When there is an increase in body fat, the adipose tissue increases the vitamin D uptake and storage, especially among obese individuals [19], then the amount of vitamin D circulating in the bloodstream decreases. Based on a previous systematic review, the chance of death caused by cardiovascular diseases, cancer and other causes is 16% higher when circulating vitamin D level reduces by every 10 ng/mL [58]. Therefore, body fat percentage was a better anthropometric measurement to predict for vitamin D insufficiency compared to BMI and waist circumference.

The respondents attained the recommended minimum duration of sun exposure per week as recommended, but it did not associate to their vitamin D status. The first possible explanation is that older adults have lower efficiency in synthesizing vitamin D compared to young adults due to thinner skin. The thinning of the skin results in less number of pre-cursor availability of vitamin D, 7-dehydrocholesterol to be converted into the active vitamin D after sun exposure [5,13]. Secondly, the respondents reported during the recall that most of them were exposed to sunlight either in the early morning from 7.00 am to 9.00 am or in the late evening from 5.00 pm to 7.00 pm, which is not within the optimal period. The optimal period for sun exposure starts from 10.00 am to 3.00 pm, because the ultraviolet B radiation within the sunlight has a long wavelength of 290 to 315nm. As a result, the ultraviolet B radiation needs a short period to pass through the ozone layer before landing on the earth surface, then absorbed by the human skin to synthesize vitamin D effectively [59]. Therefore, sun exposure in this context was not able to contribute to vitamin D levels among respondents in the current study.

The overall diet quality of the respondents did not contribute to vitamin D levels. However, the consumption of milk and dairy products was associated with vitamin D status among the respondents when evaluating the intake of individual food groups. These results are supported by previous local interventions among older women that reported that milk supplementation significantly improved their mean level of serum 25(OH)D [51,60]. Milk and dairy products currently sold within the market are usually fortified with vitamin D to improve the intake of vitamin D among the people [15]. Milk and dairy products are considered affordable and accessible alternatives to improve the intake of vitamin D compared to natural foods rich in vitamin D, such as salmon, mackerel and tuna [61]. Hence, older women are advised to follow the recommendation of one to three servings of milk and dairy products per day according to their respective energy requirement [18]. The fat content in either whole or skimmed milk does not influence the vitamin D bioavailability [62], so older women are allowed to choose any milk based on their taste preference and health condition.

Other than milk and dairy products, other food products are fortified with vitamin D, such as breakfast cereal, bread, flour and fruit juice. The fortification of micronutrients in Malaysia is monitored by the Food Safety and Quality Division [63]. Therefore, the food manufacturer is required to meet the minimum amount of nutrients to be able to claim as being fortified with vitamin D. These food products are widely available in the market and highly accessible by Malaysians with an affordable price.

The multivariate analysis reported that 6.6% of the variances in vitamin D status of the respondents could be explained by body fat percentage and number of servings of milk and dairy products consumed. The result suggested that high body fat percentage contributed the most towards insufficient vitamin D, followed by low consumption of milk and dairy products. A study conducted by Vitezova et al. [23] also reported that body fat percentage had the strongest association on the vitamin D level of older women. Previous reviews by Cashman [15] and Lips et al. [8] also support that milk and dairy products consumption can improve vitamin D status via the calcium content and vitamin D fortification.

The r-squared value obtained in the current study was low, which might be due to missing key variables, such as ethnicity and skin pigmentation. According to a previous study, Chinese females had higher serum 25(OH)D level compared to Malay and Indian counterparts in Malaysia due to their clothing styles, usage of umbrellas and sunblock [25]. Besides, the darker skin colour of Malay and Indian, which consists of higher melanin content inhibits the synthesis of vitamin D [53]. Therefore, future research related to vitamin D should consider both important variables.

The present study has a few limitations. Firstly, the current study involved only Chinese older women, whereby the results do not represent the multi-ethnicity population in Malaysia. However, the outcome of the current study is still able to provide an overview of the prevalence of vitamin D deficiency and its associated factors among the Chinese older females in Malaysia. Secondly, the sun exposure and dietary intake data obtained may not be accurate due to recall bias of the respondents in recalling their sun exposure and food intake for the past month. There was no selection bias in terms of the affiliates as the affiliates did not provide both their verbal and written consent to participate in the current study. Thirdly, the HEI for Malaysians did not take into account overconsumption of food groups as the score of 10 was given whenever the respondents achieved a minimum serving for each food group. Next, the method used for vitamin D quantification is not the gold standard, which should be the Liquid Chromatography-Tandem Mass Spectrometry (LC-MS/MS). However, the ability of the immunoassay used in the current study is comparable to the gold standard [64], so the results are reliable to be utilised.

Besides, the body fat percentage of the respondents were estimated through an equation being programmed in the body fat analyser used in the present study while reference of the equation was not stated clearly in the body fat analyser. Yet, the results of body fat percentage obtained remain valid and reliable as the device applies the established and valid bio-electrical impedance method, which have been used by previous studies [65–67]. Next, the current study did not assess the UV index. While Malaysia has a high UV index (>8), there is a possibility that the respondents avoid going outside of the house during midday. It should be noted that WHO recommends the public to avoid being outside during midday hours, seek for shade, while shirt, sunscreen, and hat must be worn in order to avoid the potential danger of sun exposure, such as sunburn. In the present study, the sun exposure data can be utilised as a proxy to understand the behavioural pattern of the respondents that lead to differences in vitamin D concentration in their body. Lastly, the nature of the present study did not determine the causal effect of body composition, sun exposure, diet quality and vitamin D status among the respondents. As of this date, this is the first study to determine body composition, sun exposure and diet quality with vitamin D status among postmenopausal Chinese women in Malaysia. Hence, the study findings can be utilized as a starting point for future research and intervention related to vitamin D.

## Conclusion

In this study, more than two-thirds of postmenopausal Chinese women had insufficient vitamin D. High body fat percentage and low consumption of milk and dairy products were associated with insufficient vitamin D levels among postmenopausal Chinese women. Hence, postmenopausal women should maintain their percentage of body fat within an acceptable range and consume the recommended amounts of milk and dairy products daily to ensure sufficient levels of serum vitamin D.

## Supporting information

S1 Raw data (XLSX)
